# BOARD-FTD-PACC: a graphical user interface for the synaptic and cross-frequency analysis derived from neural signals

**DOI:** 10.1186/s40708-023-00191-x

**Published:** 2023-05-08

**Authors:** Cécile Gauthier-Umaña, Mario Valderrama, Alejandro Múnera, Mauricio O. Nava-Mesa

**Affiliations:** 1 Grupo de Investigación en Neurociencias (NeURos), Centro de Neurociencias Neurovitae-UR, Instituto de Medicina Traslacional (IMT), Escuela de Medicina y Ciencias de la Salud, Universidad del Rosario, Bogotá, Colombia; 2 Department of Systems Engineering, Pontificia Universidad Javeriana, Bogota, Colombia; 3 Department of Biomedical Engineering, Universidad de Los Andes, Bogotá, Colombia; 4 Behavioral Neurophysiology Laboratory, Physiological Sciences Department, School of Medicine, Universidad Nacional de Colombia, Bogotá, Colombia

**Keywords:** Cross-frequency analysis, Synaptic analysis, Event-related potentials, Phase–amplitude coupling, Phase coherence

## Abstract

In order to understand the link between brain functional states and behavioral/cognitive processes, the information carried in neural oscillations can be retrieved using different analytic techniques. Processing these different bio-signals is a complex, time-consuming, and often non-automatized process that requires customization, due to the type of signal acquired, acquisition method implemented, and the objectives of each individual research group. To this end, a new graphical user interface (GUI), named BOARD-FTD-PACC, was developed and designed to facilitate the visualization, quantification, and analysis of neurophysiological recordings. BOARD-FTD-PACC provides different and customizable tools that facilitate the task of analyzing post-synaptic activity and complex neural oscillatory data, mainly cross-frequency analysis. It is a flexible and user-friendly software that can be used by a wide range of users to extract valuable information from neurophysiological signals such as phase–amplitude coupling and relative power spectral density, among others. BOARD-FTD-PACC allows researchers to select, in the same open-source GUI, different approaches and techniques that will help promote a better understanding of synaptic and oscillatory activity in specific brain structures with or without stimulation.

**Supplementary Information:**

The online version contains supplementary material available at 10.1186/s40708-023-00191-x.

## Introduction

Starting with the first electroencephalographic (EEG) recordings by Hans Berger [1], brain activity, in different oscillation bands, has been associated with cognitive processes. Since then, advances in neurophysiological techniques, acquisition methods, and signal processing (i.e., computational capability improvements) have yielded major insights into the function of specific brain structures and their relation with behavior and cognitive processes [2]. For instance, theta, alpha, and gamma band interactions in the neocortex and hippocampus are linked with memory processes [3], while beta oscillations have been related to sensory-motor activity [4].

Interactions between brain oscillators can be estimated through different methods. In this sense, cross-frequency coupling (CFC) analysis characterizes the interaction between oscillations across different frequency bands; different types of CFC have been described, including phase-to-phase (PPC), amplitude-to-amplitude (AAC), and phase-to-amplitude (PAC). In this work, we focused on two types of coupling: phase coherence (PC) [5] and PAC. There are different methods for measuring PAC: envelope-to-signal correlation [6], phase-locked measurement [7], modulation index [8], general linear model measurement [9], the amplitude of the power spectral density (PSD) [10], Kullback–Leibler-based modulation index [11] and event-related phase–amplitude coupling [12]. Each method has different advantages and limitations and should be chosen according to the data acquired and the specific experiment performed. In the case of PC, relations between two signals can be observed by their instantaneous phase difference [13]. These moments of coherence reveal an effective interaction between different areas of the brain [14] and a flexible communication structure [15]. This mechanism has been linked with several functions, such as memory [16] and attention [17].

Analysis of neurophysiological recordings may require different methodological approaches with diverse complexity levels, ranging from amplitude and latency quantification in single-cell records to the analysis of coordinated network activity of several hundred thousand active neurons (large-scale brain networks). Implementing advanced analytical procedures requires proficient management of complex mathematical methods and programming skills, which are not mastered by many neuroscientists or clinicians; it could therefore be profitable to offer them a user-friendly software application subserving such analytical methods. Although there are, in fact, some available applications and tools to analyze neural signals, some of them have been designed for particular purposes, and, therefore, they can be not easily customizable, expensive, not available for the general public or too complicated for general use. The following are some of the many other programs that have been developed for cross-frequency analysis, and which take different approaches. EEGlab [18] is a very complete and wide-ranging MATLAB toolbox oriented to scalp EEG signals, developed for event-related potential (ERP) and independent component analysis (ICA). EEGLab has a toolbox, event-related PACTools [19], that allows the use of several PAC methods. In addition to EEGLab, other software like Fieldtrip [20], Brainstorm [21], MNE [22] and Tensorpac [23], are able to estimate PC, PAC and other types of time–frequency analysis. However, some of them require advanced programming abilities, non-include a graphical interface, not allow automatic segmentation or implement only one method of PAC estimation. On the other hand, there are a lot of programs for analyzing synaptic activity such as Neuromatic [24], MiniAnalysis (Synaptosoft, Decatur, US), Minhee Analysis Package [25], Easy Electrophysiology v2.3 (Easy Electrophysiology Ltd., UK), pClamp 11 (Molecular Devices) and WCP Strathclyde Electrophysiology Software, which focus on detecting and quantifying post-synaptic events, action potential analysis and filtering, among many other functions. Few of them have tools for power spectral analysis such as Fast Fourier Transform (FFT) or Power Spectral Density (PSD), and none have specific tools for cross-frequency analysis.

For that reason, we developed BOARD-FTD-PACC (Brain Oscillations Analysis and Resourceful Display in Frequency and Time Domains Plus Phase Amplitude Coupling and Coherence) using MATLAB (The Mathworks, Inc.), integrating several analytical procedures in a single and adjustable interface to offer a tool for neurophysiological analysis of diverse intracellular and extracellular recordings including synaptic and large-scale networks analysis, that is accessible, user-friendly, practical in diverse experimental setups, and reliable. At difference of most cross-frequency analyzing programs, BOARD-FTD-PACC may detect and analyze post-synaptic events and also, includes three methods for PAC quantification: Mean Vector Length Modulation Index, Kullback–Leibler Modulation Index and Phase-Locking Value. In addition, the huge variability in experimental procedures makes it difficult to have an analysis software that can fit many different recording conditions such as electrode location and number, stimulation protocols, time window selection and trial averaging. The present software interface provides both numerical and graphical outputs from different analytical methods, allowing the user to display them either time- (average) or phase-locked (cross-frequency coupling). BOARD-FTD-PACC is of particular use in assisting researchers in cross-frequency analysis, reducing the time required to perform such analyses in different experimental settings. In the present work, we will show some examples of recordings derived from in vivo experiments in the thalamus, cortex, and hippocampus, illustrating the use of the BOARD-FTD-PACC tool for their analysis in time-, frequency- and time–frequency domains.

## Methods

Experimental data analysis often entails finding a suitable best-fit technique to extract significant information and present it in an intuitive and straightforward way. Due to the complex nature of neural signals, it has been necessary to pick up and customize a range of analytical techniques developed in mathematics, engineering, and data science over recent decades. Since there is a need for different tools for the visualization and analysis of neural signals in a research laboratory applying countless experimental setups, as well as variations in the recording duration, stimulation, and conditions of the subjects, we have selected and optimized a set of different analytical and visualization tools.

BOARD-FTD-PACC is a software application developed for the analysis of neural oscillations using MATLAB’s *App Designer*, a high-level computing environment and programming language widely used in neuroscience. Considering that MATLAB offers several advantages over other languages, that software was designed to work with linear data structures, such as the matrices used in this instance, and it is a higher-level language that provides design tools that allow apps and interfaces to be designed and used in a very intuitive way. BOARD-FTD-PACC was designed to be adaptable and intuitive, aimed at users with diverse computational backgrounds. It is intended as a semi-automatic tool, in which the users can adjust parameters such as time and frequency limits, or trial selection, according to the experimental design and analytical requirements, in order to obtain a more robust analysis. This software enables the use of different techniques to extract more information hidden in regional brain oscillations. Since the code is written exclusively in MATLAB, no external functions or libraries are required. The toolbox can be used on OS X, Linux, and Windows architectures, and installed as a MATLAB app. The output of this software is mainly independent graphs that can be saved and manipulated individually without losing previous analyses. Electrical signals produced by a large group of neurons can be analyzed in the time and frequency domains.

### Time domain

**Fig. 1:**
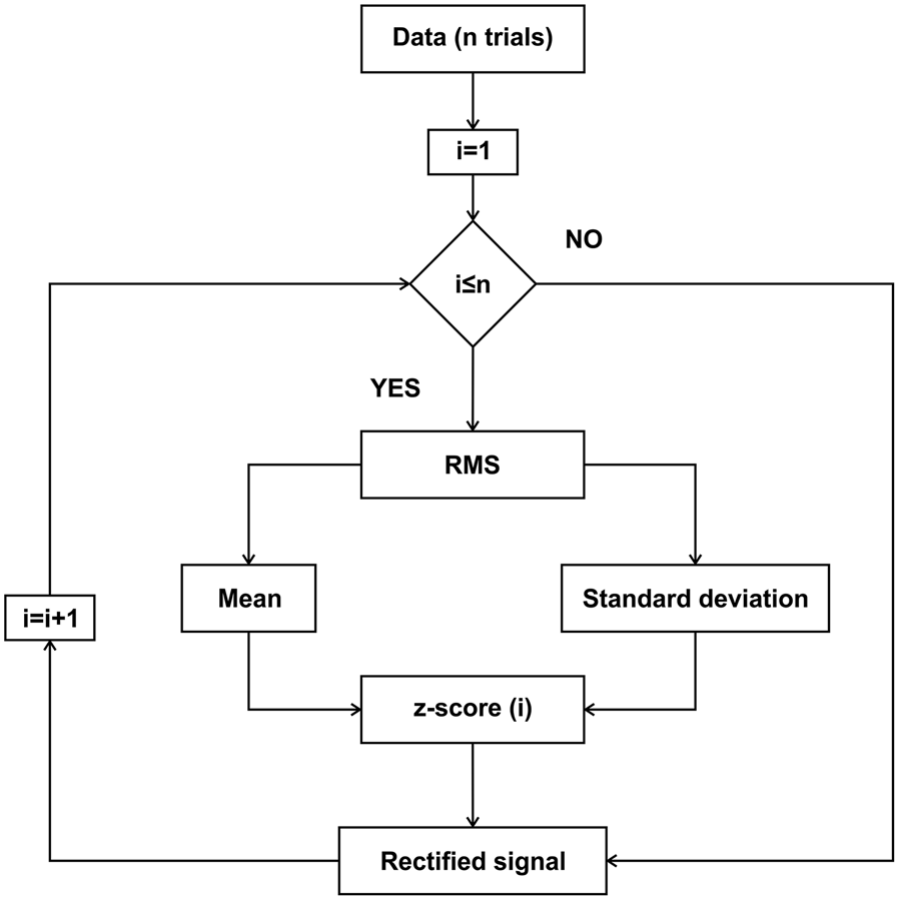
Algorithm flowchart for the implemented RMS z-score method. This method gives the neural activity relative to the mean of the signal, for every trial, as a positive number

In the time domain, some methods include (a) measuring the amplitude and latency of neurophysiological activity, (b) averaging trials, used to calculate event-related potentials (ERP), and (c) z-score visualization of the rectified signal. One of the first methods developed in the time domain was averaging, which made it possible to summarize neurophysiological recordings, even before digital computers were available [26], improving the signal-to-noise ratio [27]. Stimuli-triggered averaging of electrical activity can detect time-locked event-related responses (a time-domain graph shows how a signal changes within a selected period) with precision and accuracy. To characterize the relative changes of the mean power of the electrical activity, BOARD-FTD-PACC calculates the root mean square (RMS) of the signal and normalizes it over the mean of the raw signal using the z-score (Fig. 1) [27]. Time-locked activity can also be filtered in a single frequency band, making it possible to identify transient power changes and remove unwanted noise [28].

### Frequency domain

The Fourier transform (Ft) calculates the spectral power of every frequency from raw or filtered analog time series [29]. For discrete time series, like digitized neurophysiological recordings, it has been necessary to develop other methods based on Ft, like: (a) fast Fourier transform (FFT) [30], which analyzes discrete and limited signals; (b) Power Spectral Density (PSD), a method that estimates the distribution of power into frequency components of a signal and corrects the influence of noise [31]; and (c) relative PSD [32], which determines the percentage of energy in a specific frequency band. Such analyses rely on the presumption of stationarity, which makes them blind to dynamic changes in the power spectrum [33]. Since brain activity is temporally variable, it does not fit the stationarity criteria, thus the above-mentioned methods have limited sensitivity to transient activity changes in neural signals.

### Time–frequency domain

Time–frequency domain-based analyses are aimed to detect temporal variations of spectral power in different frequency bands to solve the limitations of pure frequency domain methods. A first approach was to iteratively calculate FFT over successively shorter time windows (with or without overlapping between them) and stack them to construct a three-dimensional display (spectrogram). However, such window size limits spectrogram time or frequency sensitivity and biases the results [34]. In 1984, Morlet and Grossman used wavelets to produce a time–frequency analysis with adjustable time windows using the number of cycles in a wavelet of a particular frequency (scalogram) [35, 36]. Within the different types of wavelets, the Morlet method is the Gaussian model of a sine wave with better results in the context of EEG signal analysis [37]. Other methods such as Choi–Williams distribution (CWD), base their analysis on the use of kernels, generally exponential functions instead of sinusoidal functions. Although it can present good results, it needs parameter adjustment to reduce artifacts, as well as to avoid aliasing. In addition, the signals analyzed by CWD must be short and it has a high computational cost [38]. The most flexible method that has a good compromise between resolution, computational cost and flexibility is the Morlet wavelet, and for that reason was implemented in BOARD-FTD-PACC.

### Phase analysis

**Fig. 2:**
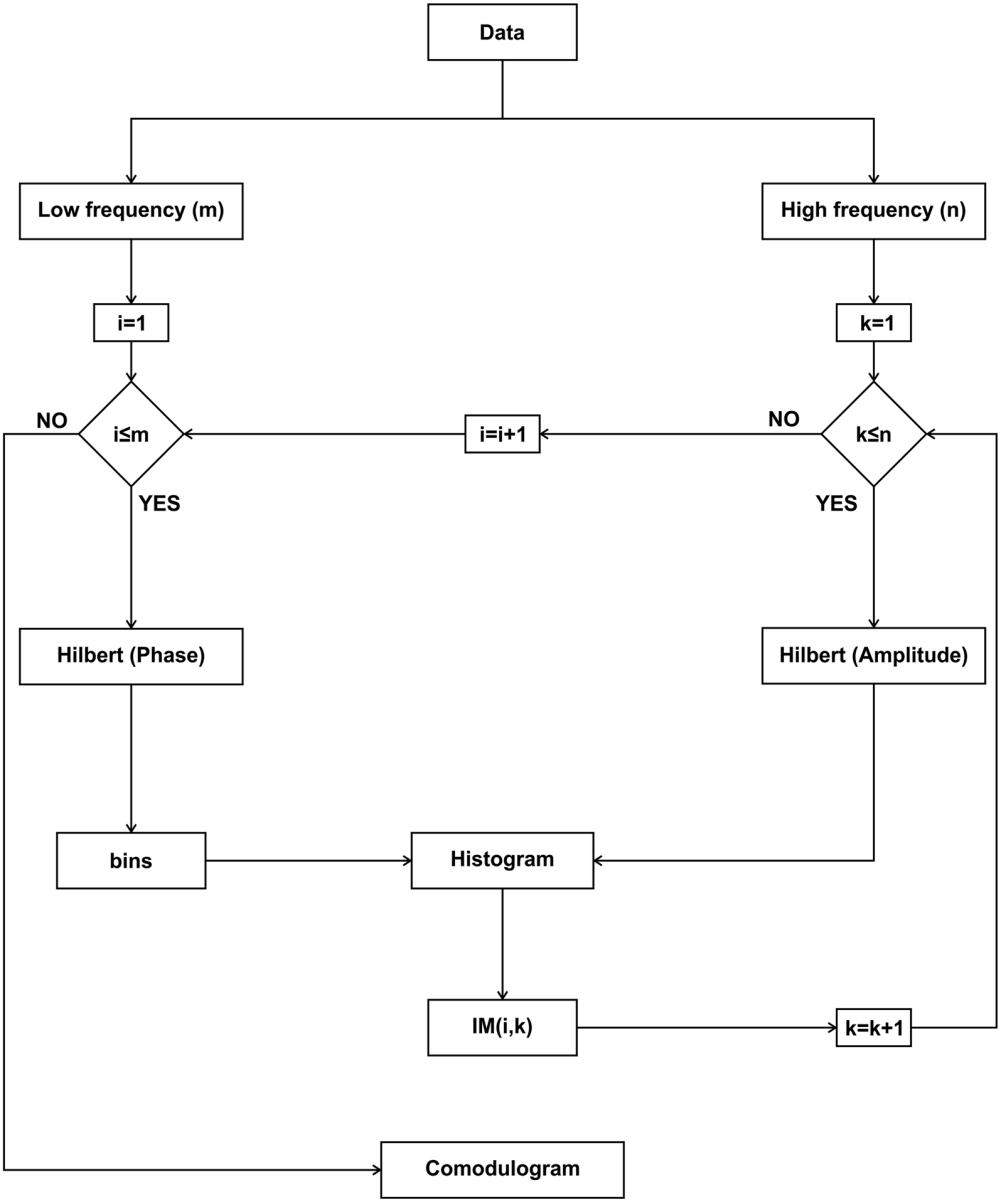
Algorithm flowchart for the implemented PAC (comodulogram) method. The steps for calculating this phase–amplitude plot can be followed for a single channel or for the interaction of two channels, by choosing the inputs as a single or two separate channels

**Fig. 3:**
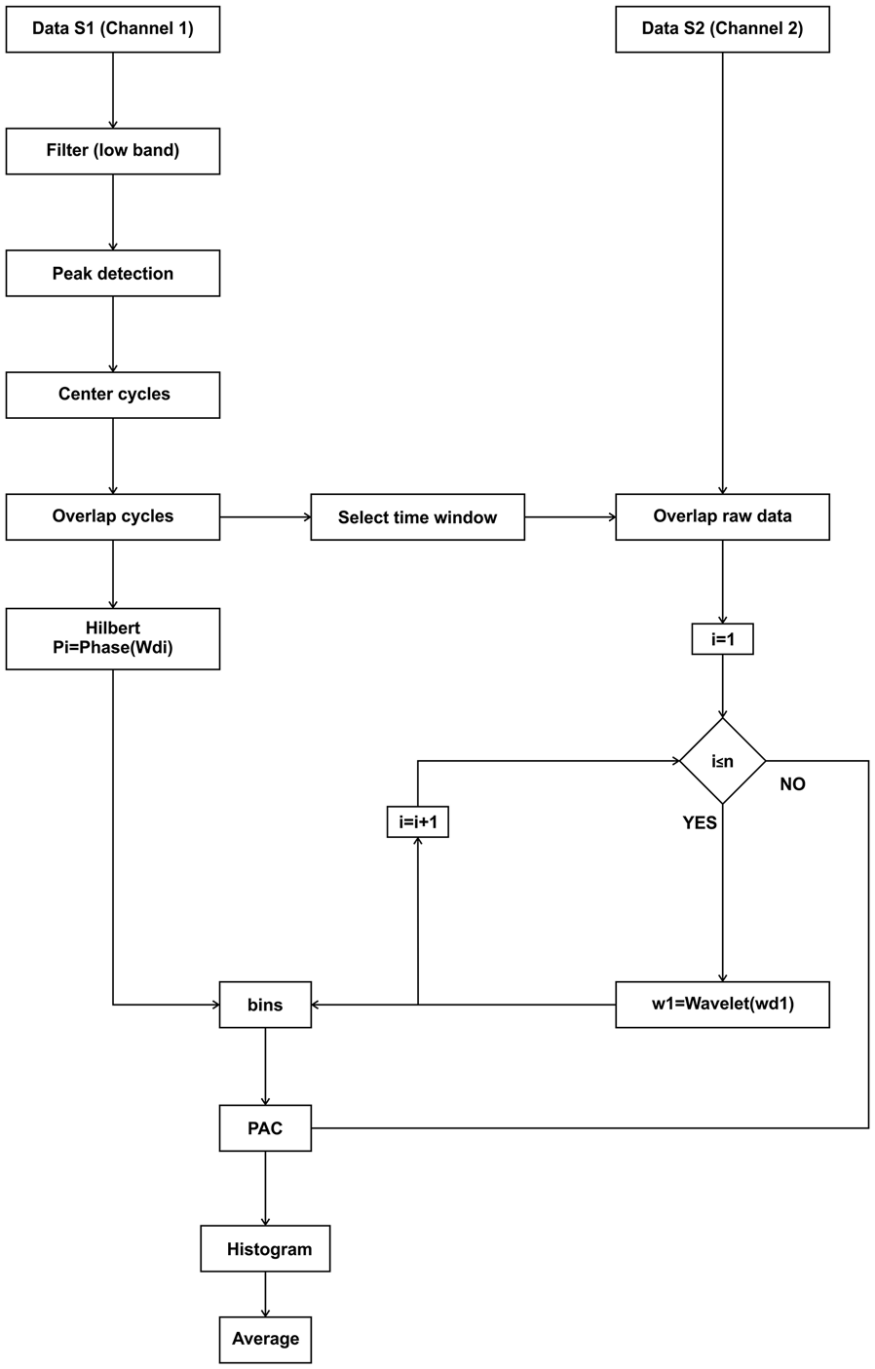
Algorithm flowchart for the implemented PAC (cycle detection) method. This method can be used to measure the modulation in a single channel or for the interaction of two channels

PAC can be used to measure modulation locally or between two brain regions, and different approaches can be used to calculate it:

1. Comodulogram-based modulation index [11] (Fig. 2). For this method, two frequency bands are selected: a narrow and low-frequency modulating band (${f}_{p}$), and a broad and high-frequency modulated band (${f}_{A}$). For each combination of these frequency windows, we determine the modulation index (MI) of that pair of frequencies. At the end, a comodulogram is plotted with the MI for each combination. The raw signal $x\left(t\right)$ is filtered in two frequency ranges (${f}_{p}$ and ${f}_{A}$), obtaining ${x}_{{f}_{p}}\left(t\right)$ and ${x}_{{f}_{A}}\left(t\right)$. The phases ${\phi }_{{f}_{p}}\left(t\right)$ of ${x}_{{f}_{p}}\left(t\right)$, as well as the amplitude ${A}_{{f}_{A}}\left(t\right)$ of ${x}_{{f}_{A}}\left(t\right)$, are calculated using the Hilbert transform. The phases ${\phi }_{{f}_{p}}\left(t\right)$ are binned and a composite phase–amplitude time series (${\phi }_{{f}_{p}}$, ${A}_{{f}_{A}}$) is calculated to obtain the mean amplitude distribution over phase bins. The MI is obtained by normalizing the average for every pair of frequency windows. These steps are repeated for each of the *m* low-frequency windows and the *n* fast-frequency windows and are represented in a *n* by *m* comodulogram **(**Fig. 2**)**.
2. Average high-frequency power over the modulating low band [39], is a variation of the previous method. Only one modulating band is analyzed, so the phases ${\phi }_{{f}_{p}}\left(t\right)$ are calculated and binned. For the faster-modulated band, time–frequency analysis is performed using a Morlet wavelet transform, ${TF}_{{f}_{A}}$. We now have a composite phase–amplitude time series (${\phi }_{{f}_{p}}$, ${TF}_{{f}_{A}}$) that allows us to obtain the mean power per phase bin. The average of the entire band can be calculated to obtain the power distribution and its mean gives a single value for the pair of frequency bands.
  This method can be also used for every trial that is being analyzed and averaged to get a general idea of the recording.
3. Average high-frequency power over the modulating low band cycles (Fig. 3). This method is especially useful when the modulating band oscillations are not continuously present across the experiment. To analyze the modulating band, after filtering, a peak detection algorithm is used to determine the local minimum of the wave; then, a time window is selected for each low-frequency band cycle $wd\left(j\right)$, and the phases ${\phi }_{{f}_{p}}\left(t\right)$ are calculated and binned. A wavelet transform ${TF}_{{f}_{A}}\left(j\right)$ is calculated for each corresponding time window of the raw modulated band A phase-power time series (${\phi }_{{f}_{p}}$, ${TF}_{{f}_{A}}$) allows us to obtain the mean power per phase bin. The power distribution is obtained by averaging the entire modulated band power for each modulating band phase, giving a single amplitude value for each phase bin.

**Fig. 4:**
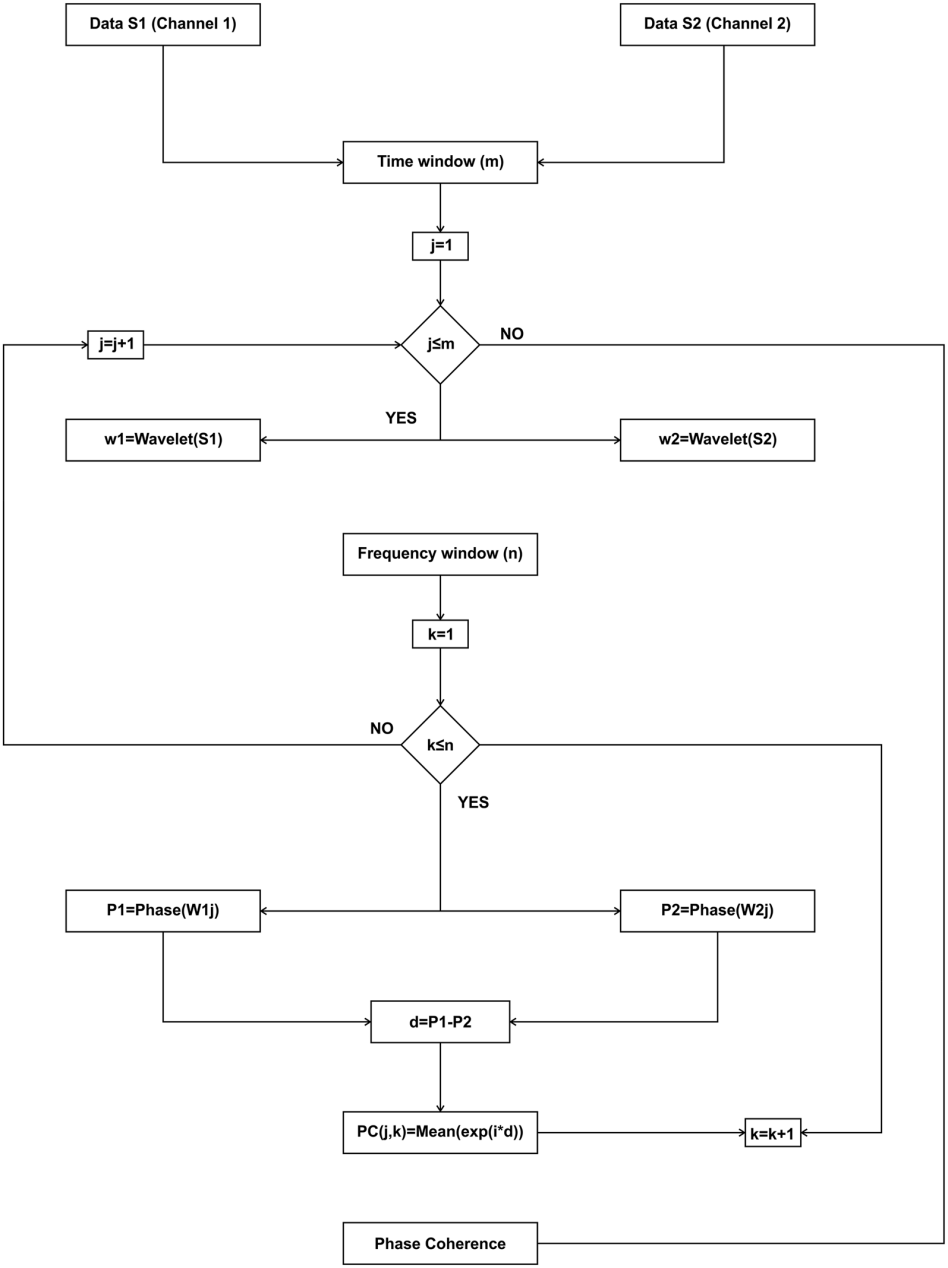
Algorithm flowchart for the implemented PPC method. For each channel ($S1$ and $S2$), *m* windows of time are determined, and the Morlet wavelet transform is calculated for each window in both channels. For each time window, *n* frequency windows are determined, and their phases are calculated. The difference between the two phases is measured for every time–frequency epoch and this is used to calculate the phase-coherence value, which is represented in an *n* by *m* matrix

PC: For phase coherence, which can only be determined between channel pairs, the same phase approach is used (Fig. 4). PC displays temporal information of phase synchrony between separated brain areas, which is indirect evidence of their functional interaction [40]. For this analysis, several time windows are selected and, in every one, Morlet wavelet transform is applied to each channel (${W}_{1}\left(j\right)$), then a frequency window is selected to obtain each channel phase (${P}_{1}\left(j\right)$ and ${P}_{2}\left(j\right)$) from the wavelet output. For every time–frequency epoch, the difference between the two phases was calculated ($d={P}_{1}\left(j\right)-{P}_{2}\left(j\right)$) and used to define the phase-coherence value $PC\left(j,k\right)=mean({e}^{id})$.

**Fig. 5:**
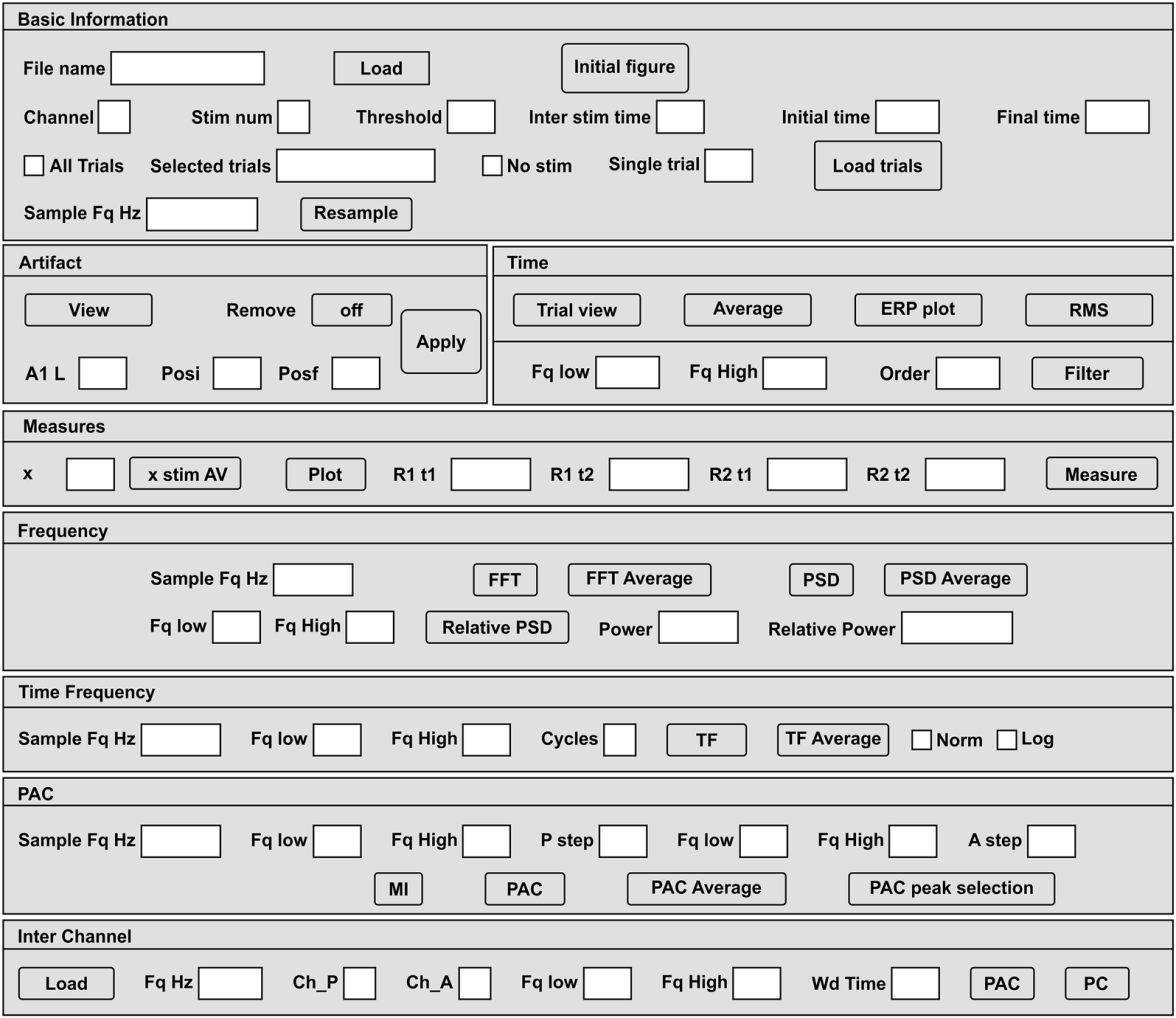
General overview of the BOARD-FTD-PACC interface

Representative intracranial recordings integrated with the flowcharts and analytic methods of figures 1-4 are included as Additional file 1 (Fig. S1–S4). Finally, an overview of the BOARD-FTD-PACC interface is presented in Fig. 5.

## Results

### Software overview

The following examples were derived from simultaneous in vivo recordings in the thalamus and cortex of anesthetized rats.

#### Initial setup

The aim of this work was to present a platform for the analysis of electrophysiological signals recorded in different experimental configurations that include different setups, with anesthetized or free-moving subjects, in chronic or acute experimental conditions, with or without neural stimulation. To illustrate the different applications, we present the images produced as examples that came from intracranial in vivo recordings from an anesthetized rat. Users start using the interface by opening the file BOARD_FTD_PACC.mlapp using MATLAB. This file opens a general window which displays the different tools and analyses that can be used. It is important to know the name of the file and the structure of the experiment since they are necessary when defining the parameters that allow the analysis to be carried out correctly. One of the strengths of BOARD-FTD-PACC is that it is a semi-automatic tool, designed to be used by someone who knows the experiment as well as the acquisition conditions. Using this interface, they can adjust the analysis conditions to their experiment accurately and not leave such decisions up to a fully automatic program. Once such parameters are set, the analysis will be automatic.

#### Data preprocessing

**Fig. 6:**
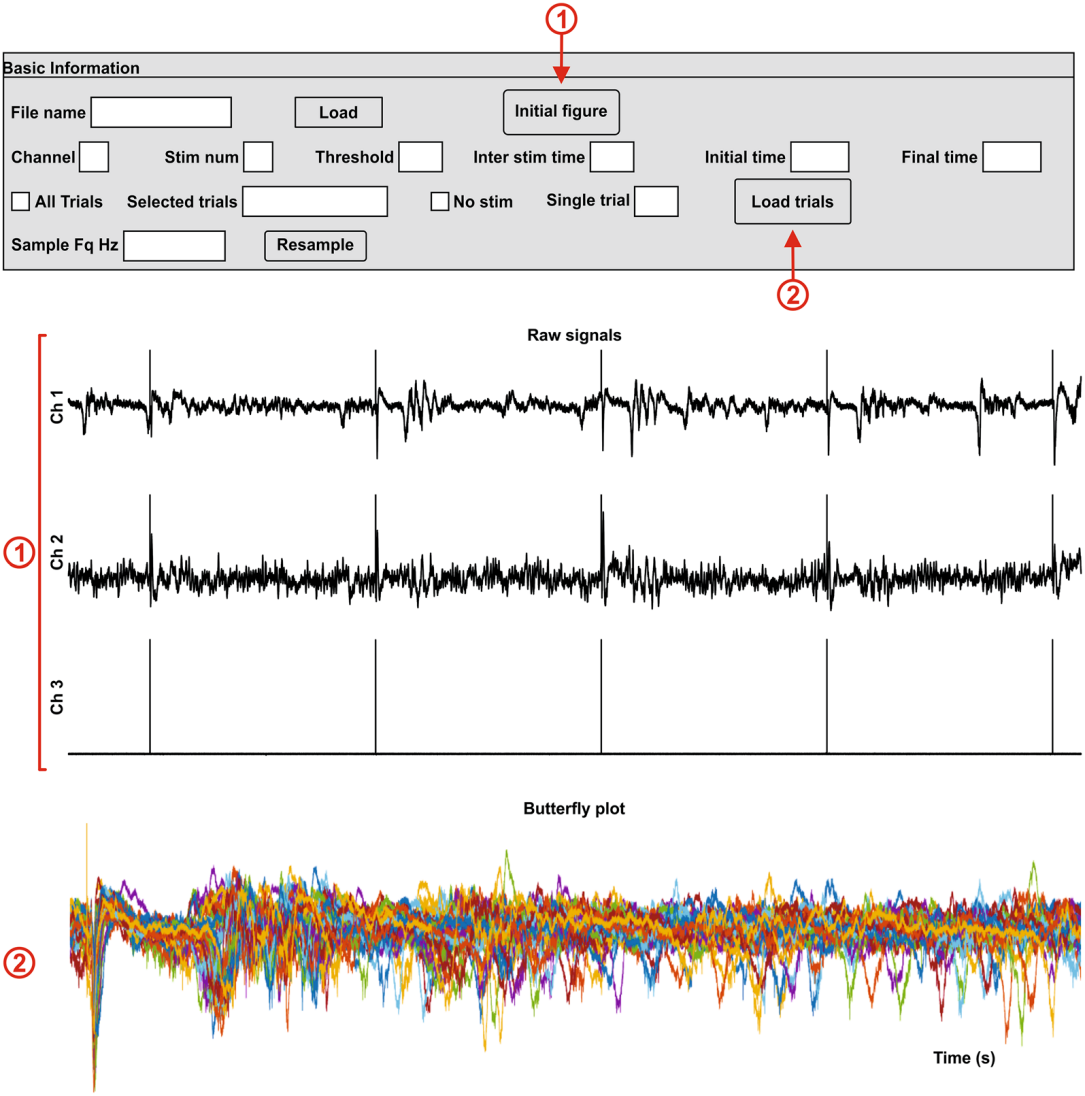
Initial setup. This section allows the user to open the signal to be analyzed in axon binary format (.abf) and displays an initial figure of the raw signal of every channel. The user can select the experiment and analysis conditions by selecting the channel, determining if the recording has stimulation (stim) or not and its number (Stim num), giving the separation or time between two stimuli (Inter stim time), and setting the amplitude (Threshold) to define trials. The user also chooses the time window (Initial and Final Time), sampling frequency (Fq) in Hertz (Hz), and trials used for the analysis. This section displays the raw signal **①** and the trials selected by the user **②**

BOARD-FTD-PACC loads digitized signals stored in axon binary file (.abf) format and the European data format (.edf). To load a file, its name must be typed in the File name box (Fig. 6), and then the Load button must be clicked (f_LoadSignal).

By clicking the Initial figure (f_Plot_Ch) button (Fig. 6①), a window showing the raw signal of every channel in the file is displayed (Fig. 6①). Then, the user can select the signal to be analyzed by typing its number in the Channel box. Whenever stimulation has been used in the experiment, the channel containing this signal must be typed in the Stim num box, and interstimulus interval (in seconds) must be typed in the Inter stim time box. If the stimulation channel is a waveform one, minimal stimulation amplitude (in mV) can be typed in the corresponding Threshold box. Then, time window limits must be set by typing values in the Initial time (since stimulus time is set as zero, initial time can either be negative or positive, Fig. 6②) and Final time boxes. The user can now select how to display the successive time windows: if all trials are to be overlaid, a tick has to be typed in the All trials box; if selected trials are to be displayed, their numbers must be listed in the Selected trials box. If no stimulation was used in the experiment, the user can tick the No Stim box, and the signal will be displayed as it was during the experiment. Whenever it is necessary, the user can downsample the signal, by typing the desired sampling frequency in the Sample Fq Hz box. Once all these parameters have been chosen, the user saves them by pressing the button Load trials (f_Select_Trials), which displays the selected time windows.

#### Time

For the time domain, five analyses are available: single trial (f_plot_single), average (f_Average), ERP plot (f_Butterfly), RMS (f_RMS_zscore) and signal measures (such as slope and latency) (f_Measure_Slope). Single-trial activity displays a figure with the raw signal for the selected trial in the basic information panel (Fig. 7①). The average shows time-locked activity in the recording (Fig. 7②). The ERP plot displays a figure with two plots: the activity from every trial overlaid in the same figure; as well as every trial activity in a matrix (*m* x *n*), where *m* is time, *n* is each trial, and the amplitude is given by color (Fig. 7③). The z-score of the RMS presents the activity relative to the mean, representing a measure of the number of standard deviations that each point of the signal is away from the mean. The final figure gives a notion of the relative activity for every trial (Fig. 7④).

**Fig. 7:**
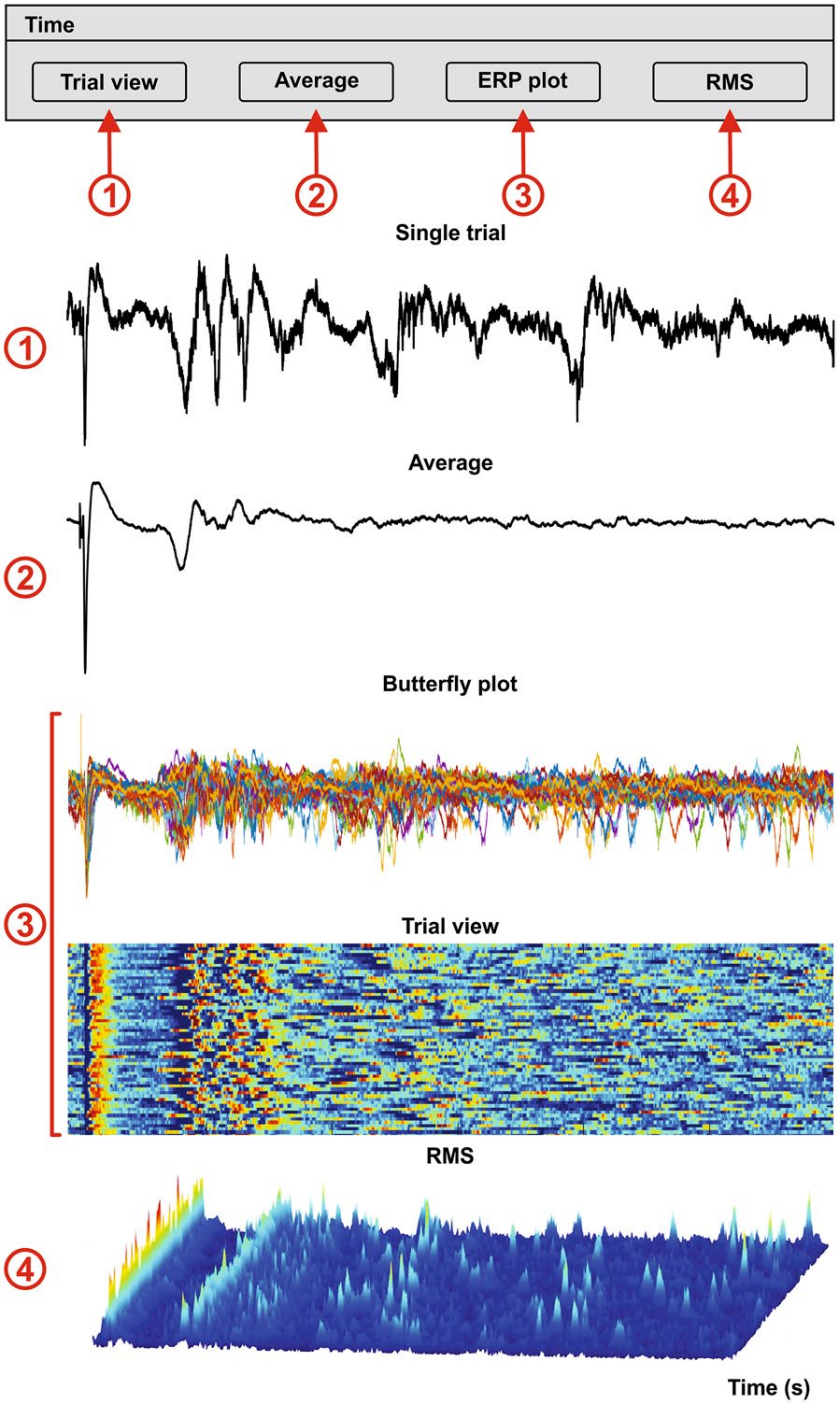
Time domain analysis. The user can display a single trial **①** the average **②**, an ERP window **③** that consists of the butterfly plot and a representation of every trial, and finally the z-score of the RMS of every trial **④**

Measures of the slope and latency of any intervals, determined by the user, can be calculated in single trials or in averages, while the number of trials selected for the average is determined by the user (x), then plotted (plot) and finally measured (Measure). For instance, we used it to analyze excitatory post-synaptic potential slopes (EPSPs) in experiments of synaptic plasticity induction (long-term potentiation/LTP). Slope measures are also used to characterize stimuli responses and spontaneous activity. EPSPs are calculated using the maximum derivative value in the time window chosen by the user, and they have the ability to customize the initial and final point for the measurements, and extend this to the different trials, allowing the user to get a very specific response, without unwanted components of the response, and without spending hours selecting each point individually.

#### Frequency domain and filtering

Displaying the activity in a defined frequency band allows us to see how it varies over time; to this end we use an adjustable Butterworth bandpass filter (ISBN-10: 0138147574). BOARD-FTD-PACC allows the user to choose the frequency limits for the Butterworth bandpass filter and its order (f_Filter_order_manual).

This section calculates the frequency response to verify the effectiveness of the filter. It opens several windows, including one that displays the original signal as well as the filtered signal and the phase for the selected trial, and another that shows the envelope and phase for every trial (Fig. 8①).

**Fig. 8:**
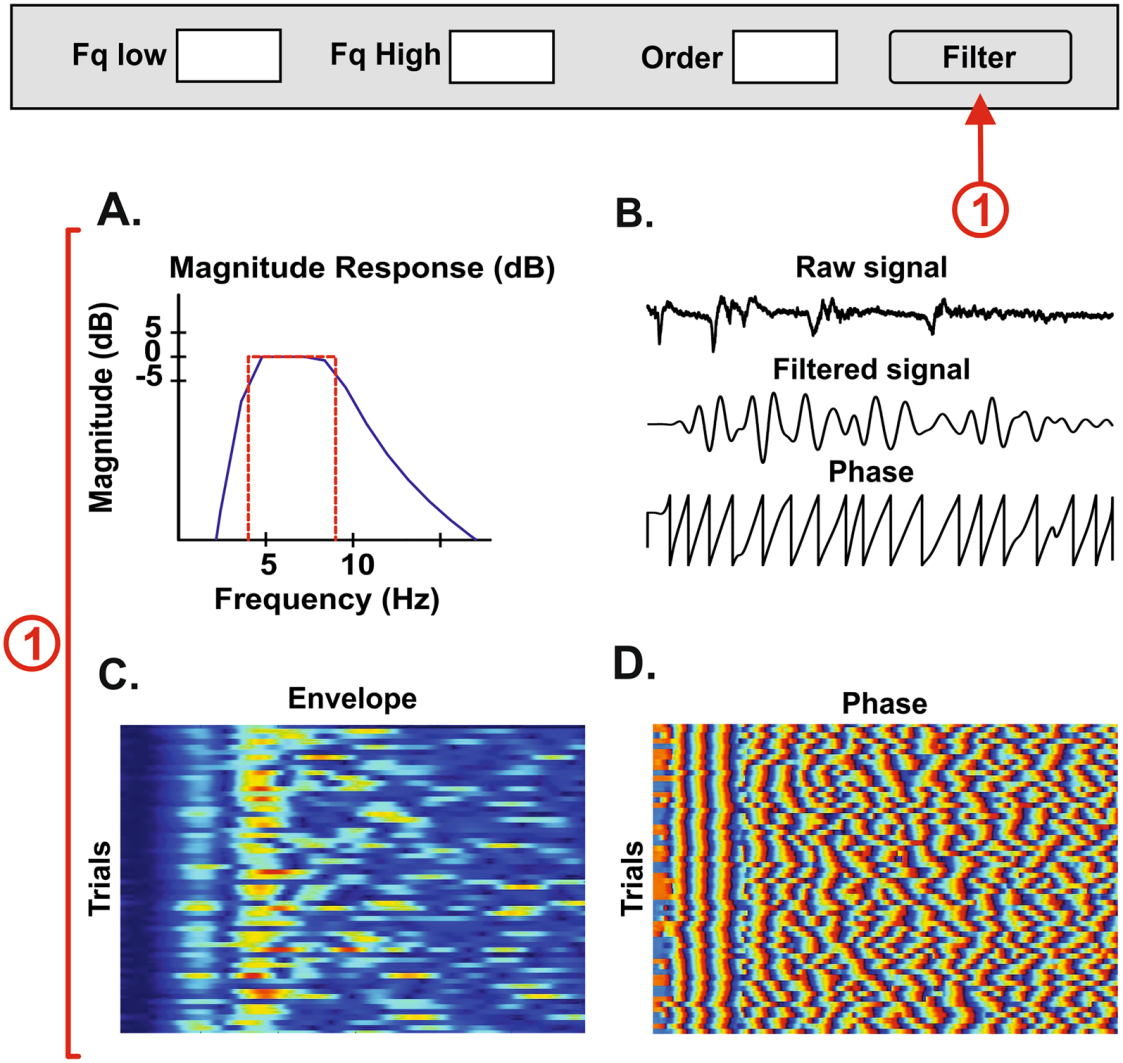
Filter. The user can determine the limits of the bandpass Butterworth filter—the low frequency limit (Fq low) and high frequency limit (Fq High)—as well as the order. BOARD-FTD-PACC displays: **A** the frequency response of the transfer function for the implemented filter (blue) vs the ideal filter (red); **B** a figure displaying the raw signal (top), the filtered signal (middle) and phase (bottom) for a selected single trial; **C** the envelope for every trial; and **D** the phase for every trial

#### Frequency domain and power

FFT allows us to know the main frequency components of the recorded data so that we can explore further the behavior around those specific bands. Another technique that gives information about the frequency content is the power spectral density (PSD), which displays the distribution of power into frequency components. In this case, we use the Welch method. These techniques give the information for the entire time window for which we do the analysis, treating the data as stationary, even though the brain is a highly dynamic system.

BOARD-FTD-PACC allows users to display the FFT (Fig. 9①) (f_FFT) and PSD (Fig. 9③) (f_PSD) of a single trial and also the average FFT of every selected trial (Fig. 9②) (f_FFT_average) as well as the average PSD (Fig. 9④) (f_PSD_Average).

**Fig. 9:**
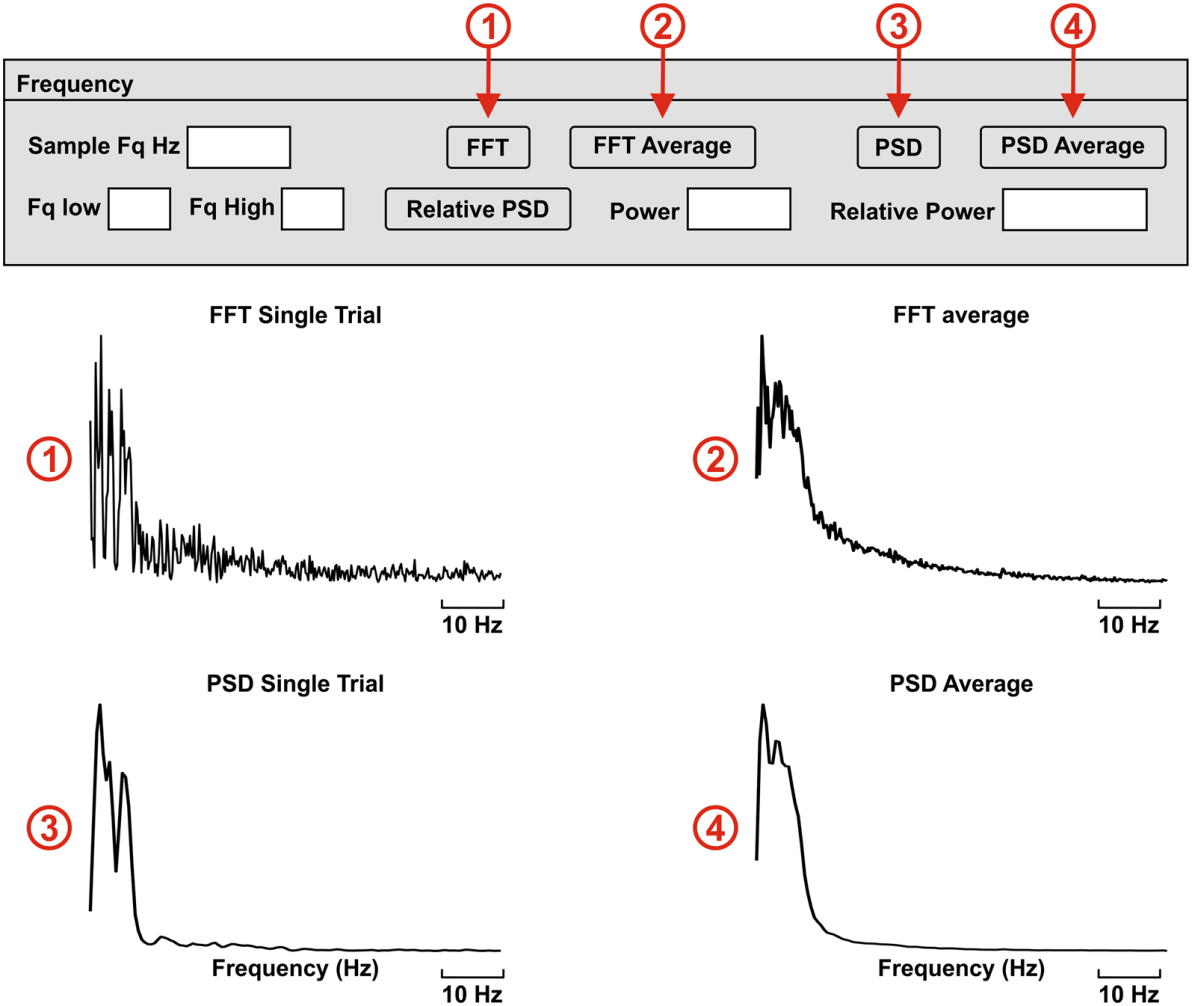
Frequency domain. This section displays the FFT of a single trial **①**, and the FFT average of all selected trials **②**, as well as the PSD of the single trial **③**, and the average PSD **④**. The PSD and relative PSD of any band are calculated by the software, while the frequency limits (Fq low to Fq high) are determined by the user. In order to accelerate the calculations, the signal can be resampled to a desired lower sample rate (sample frequency (Fq) in Hertz (Hz))

It calculates the PSD and relative PSD of a band determined by the user. This step allows us to determine the frequency bands that are to be explored with the time–frequency and cross-frequency coupling methods.

#### Time–frequency domain

Wavelet analysis is one of the most powerful time–frequency analyses that can be done to visualize the changes of spectral power over time. There are different wavelets that meet these requirements, but we use the Morlet function.

BOARD-FTD-PACC allows the user to choose the frequency limits for the calculation of the time–frequency transform using the Morlet wavelet for a single trial (Fig. 10①) (f_tf), as well as the average (Fig. 10②) (f_tf_average). Being an adaptable software, normalization (Fig. 10③) and logarithmic scale (Fig. 10④) are options to adjust to the variations of different recordings.

**Fig. 10:**
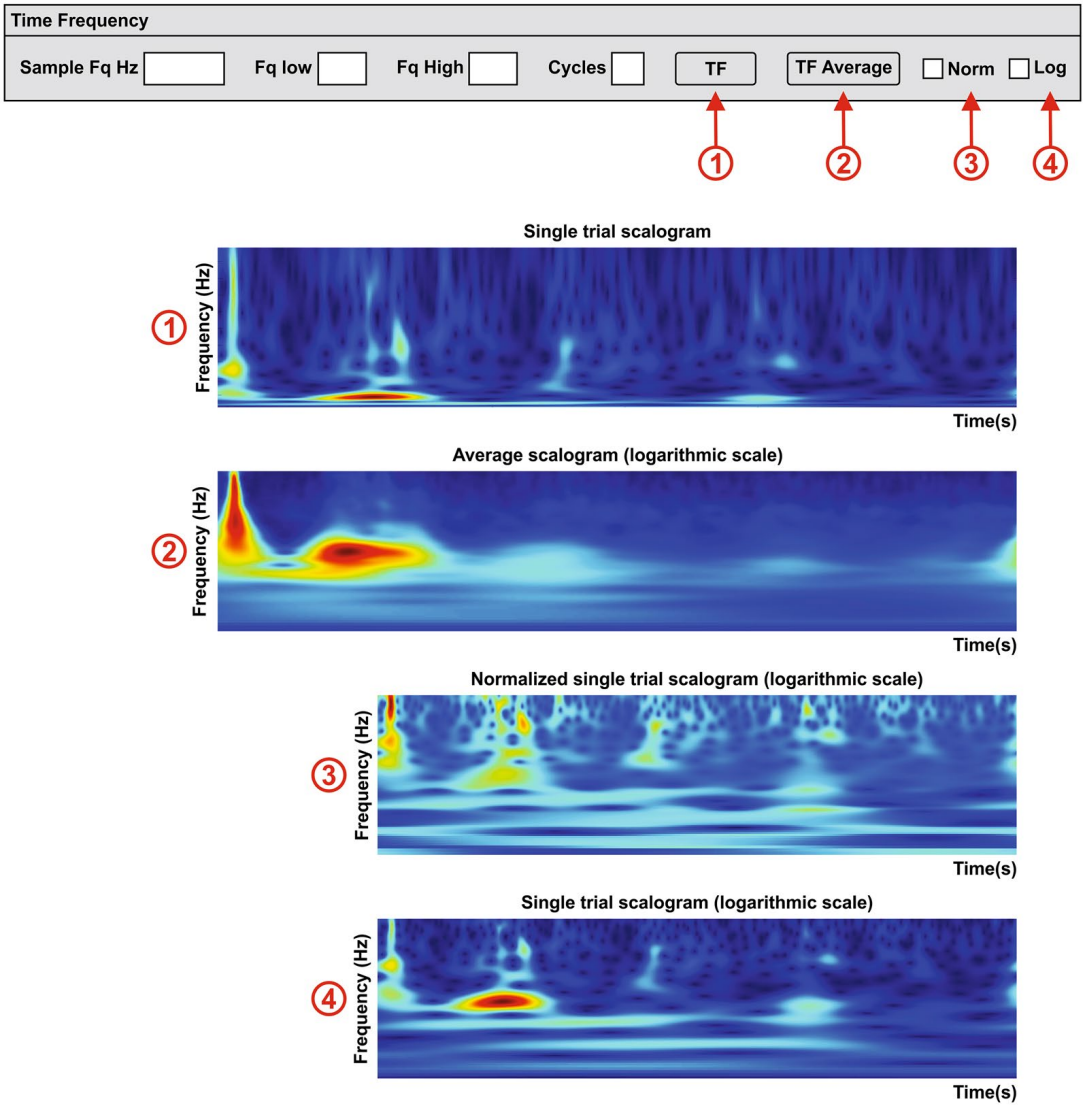
Time–frequency domain. This section displays time–frequency wavelet transform (scalogram) in the frequency limits (Fq low to Fq high) using a determined cycle number, determined by the user. It includes the time frequency (TF) of a single trial **①** and the average **②**. To better visualize the results, the user can choose the normalization (Norm) option **③** and/or a logarithmic (Log) scale **④**. In order to accelerate the calculations, the signal can be resampled to a desired lower sample rate (sample frequency (Fq) in Hertz (Hz))

#### Cross-frequency coupling

There are different types of cross-frequency coupling. In this GUI we focused on two: phase–amplitude (PAC) and phase-to-phase (PPC) coupling.

The main goal of this software was to calculate the frequency coupling. It allows the user to choose the different frequency windows and limits as well as the modulating channel. Different types of recordings demand different types of methods. For a single-channel approach, a first exploration can be done with a comodulogram (Fig. 11①) (f_PAC_sing), since this method works best with continuous or medium-length signals. For longer signals, the high-frequency power over the modulating low band method can be used for the average of various trials or to display the result for a single trial (Fig. 11②) (f_Phase_PAC) and the average over the entire recording (Fig. 11③) (f_Phase_PAC_Average). For recordings where the presence of the modulating frequency is not constant, the average high-frequency power over the modulating low band cycles method is highly recommended (Fig. 11④) (f_Phase_PAC_Pdetect).

**Fig. 11:**
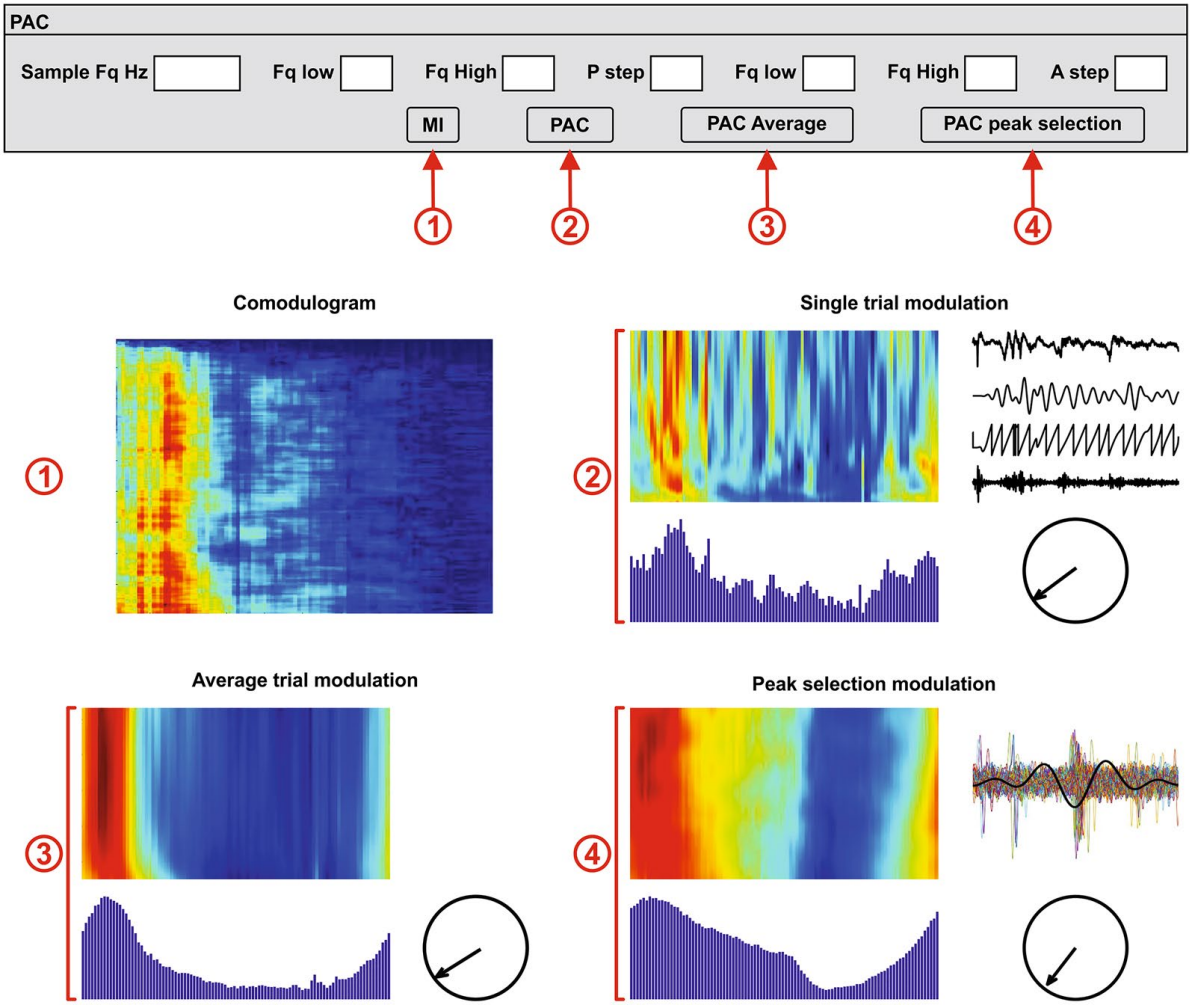
Phase–amplitude coupling of a single channel. The user can determine the high and low-frequency bands (Fq Low and Fq High), as well as the frequency windows, P step for the slow modulating frequency (phase) and A step for the fast-modulated frequency (amplitude). This section displays the comodulogram **①**, the PAC of a single channel, which can be calculated for a single trial **②**, and the average PAC for every trial **③**, using the average high-frequency power over the modulating low band method, and of the entire recording **④** using the average high-frequency power over the modulating low band cycles method. In order to accelerate the calculations, the signal can be resampled to a desired lower sample rate [sample frequency (Fq) in Hertz (Hz)]

In order to determine the interaction between two areas of the brain, the PAC can be calculated using the average high-frequency power over the modulating low band cycles method (Fig. 12①) (f_Phase_PAC_Pdetect_inter), and the phase coherence method (f_Phase_coherence).

**Fig. 12:**
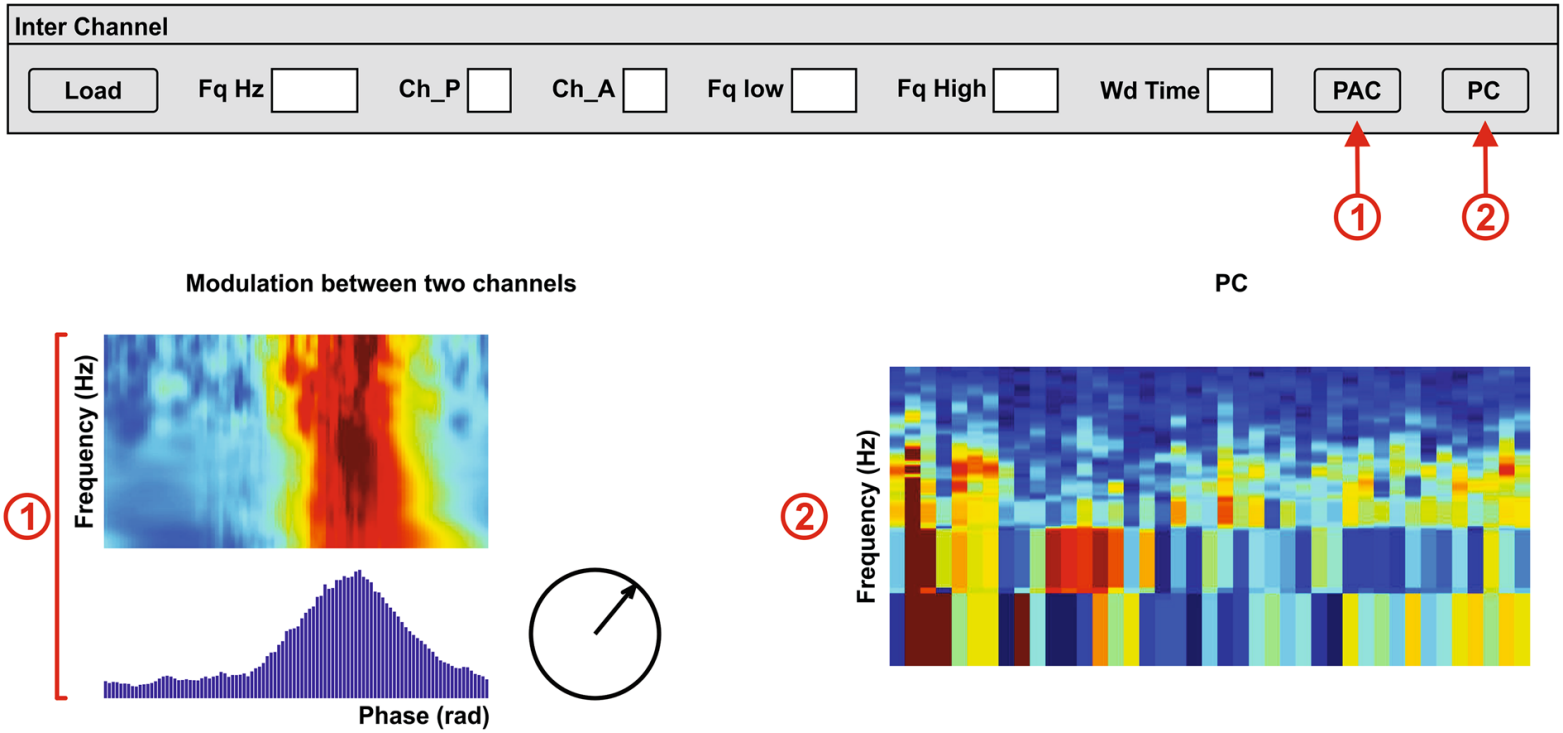
Cross-frequency coupling between two channels. This section displays the interaction between two channels. The user determines the sample rate (Fq Hz), the modulating channel (Ch_P) and the modulated channel (Ch_A), as well as the frequency limits (Fq low to Fq high) and the time window for the phase coherence analysis (Wd Time). The interface calculates the PAC using the peak selection method (Fig. 3) **①**, and the single-phase coherence (PC) **②**. In order to accelerate the calculations, the signal can be resampled to a desired lower sample rate [sample frequency (Fq) in Hertz (Hz)]

## Discussion

In the present work, a new tool is presented for neurophysiological signal processing derived from recordings in different setups, whose strength is its ability to adapt. BOARD-FTD-PACC is designed to analyze either single-channel data (temporal, frequency anchored in time, frequency, and modulation) or interactions between two channels (coherence and modulation), but leaves the parameter setting to the researcher so that they can control the scope of the analysis, as well as including a rapid system for graphical representation and several types of analysis in the frequency and time domains. It was intended as a user-friendly semi-automatic tool, to be used by researchers with or without computer science and mathematical backgrounds, and it enables the display of information extracted from different analyses made of neurophysiological recordings obtained through widely variable experimental designs. Several analytic techniques in different time and/or frequency domains were implemented, such as average, ERP display, z-score, filtering, FFT, PSD, wavelet analysis, and PPC. Such techniques are suitable to be applied one or two channels at a time, and are easily adjustable according to the type of experiments performed in a neurophysiology laboratory. Another main feature is the control the users have over the analytic procedure, regarding parameter selection and choice of temporal limits, thereby allowing a full understanding of the results and their context.

Rhythmic variations in brain field potentials in different frequency bands depend on multi-level and cross-modulating interactions between local and/or distributed oscillators [41, 42]. Such interactions may be physiological mechanisms for neural computations and inter-areal communication [43]. In fact, low-frequency rhythms (e.g., theta) engage larger brain areas and modulate spatially localized fast (e.g., gamma) oscillations [44, 45], a mechanism that is relevant to encoding and retrieving memory traces through theta-to-gamma coupling [43, 46, 47]. We presented some examples where BOARD-FTD-PACC software may represent in the same graphical interface the relationship between oscillatory activity, modulation between frequency bands and synaptic activity across the time. In order to obtain high-resolution time–frequency representations of the neurophysiological signals, spectral analysis techniques (i.e., FFT) are usually incorporated in a fixed time window. Since frequency bands and time windows may vary across an experiment, the wavelet transform was implemented in BOARD-FTD-PACC to determine a scalogram over the spectrogram. With the Morlet method a window size varies according to the frequency changes, improving the resolution of the graphical representation [33]. In comparison with Wavelet transform, quadratic time–frequency distribution methods (i.e., Choi–Williams distribution—CWD) are constant to time and frequency shifts [48, 49], and therefore, it has been considered more precise for EEG-based detection [50]. However, myoelectric signals during dynamic activity are better estimated by the continuous wavelet transform in comparison with the short-time Fourier transform, the Wigner–Ville distribution and the CWD [51]. Further studies should assess those analytic methods or combination of methods to be implemented according to the circuit studied, their physiological function, specific oscillatory characteristics, stationary Vs nonstationary neural signals and the its dynamics in a specific cognitive performance.

As we mentioned before, some other programs like EEGLab [18], Extended Modulation Index toolbox [52], MEAnalyser [53], ERPlab [54], ERPWAVELAB [55], Brainstorm [21] and FieldTrip [20], as well as libraries and tool boxes such as EMEGS [56] or PyEEG [57], have been developed for similar proposes but without an integrated interface for synaptic, power spectral and cross-frequency analysis. Other software like ERPwavelab [55] focuses on multichannel time–frequency analysis of EEG and magnetoencephalography (MEG) event-related activity, but its analytical range is limited due to its emphasis on inter-channel interactions. RIPPLELAB [58] was designed exclusively for the detection, analysis, and classification of high-frequency oscillations (HFO). ERPLAb [54], a tool integrated with EEGLab, was designed specifically to analyze ERPs, showing the activity in all the electrodes simultaneously, and making it possible to localize the source of every ERP component; it analyzes the relation between many channels instead of performing different analyses on each individual channel. MEAnalyser is a tool focus on spike train analysis with the advantage of include statistical and functional connectivity analysis, as well as graphical user interface [53].

BOARD-FTD-PACC uses three different methods for PAC analysis to obtain a better numeric estimation. In order to discriminate spurious from authentic PAC, recently was developed a tool for PAC detection based on the measure of extended MI [52]. According to the same authors, that tool has some problems in the automatic assignment of coupling origins and also, the extended MI was designed only for analysis of augmentations. In addition, that tool is focused only in PAC detection at the difference of BOARD-FTD-PACC, which also includes PC and PSD analysis. In a recent development, a nonparametric multitaper estimator of PAC was proposed to solve some pitfalls in cross-frequency statistical analysis [59].

When analyzing neurophysiological signals, the experimenter faces the option of using an interface, which generally makes decisions such as defining thresholds automatically, but analyzes the signals more easily and efficiently. These programs generally do not allow the user to define parameters that vary widely between different experimental designs, and which can be easily defined visually, such as analysis times, thresholds, selection of components within the same response, number of stimuli, or even the method that they want to use for the same type of analysis. Another option, if the experimenter is proficient in programming and mathematical analysis, is that they can develop programs that fit only their signals, but they must repeat this process each time different approaches are used. It is also very common that the experimenter does not know how to program the methods adjusted to their experiment, which can push them to carry out measurements manually so as not to lose control of their analysis. This third option has a high cost in time and energy, which limits the number of analyses that can be performed, leading to loss of information.

Most of the available toolboxes receive data exclusively in a specific format and do not adapt to different experimental data structures, such as changes in stimuli number or interstimulus interval. By contrast, BOARD-FTD-PACC allows the study of recordings while taking in account such variations. It is designed specifically for researchers that want to implement different methods in a specific and unique way. Although published works [11, 60] using some of the methods implemented here include the code required, its specific implementation for a given experimental setup requires some degree of computational and/or mathematical expertise, which makes it extremely difficult for non-expert users to include them in their studies. The approach of BOARD-FTD-PACC is to leave the decisions of the analysis parameters to the researcher, who can introduce the specific experimental setup. For instance, DC levels can be removed to eliminate DC-related errors, artifacts can be removed manually, times and trials to be analyzed can be selected, band filtering cutoff frequencies can be manually adjusted and the sample rate can be varied according to analytical requirements. This approach is an advantage as well as a limitation: the user must know and understand the structure of the experiment or else the settings will not make sense and the interface will not produce adequate results. BOARD-FTD-PACC incorporates a wide variety of analyses, which the user must be familiar with in order to understand the results provided, and to determine the correct settings. We hope it will increase the information obtained from neural activity and encourage researchers of different backgrounds to test different methods and analytical approaches that they might not have considered before.

## Supplementary Information

**Additional file 1:** Figure S1. Algorithm flowchart for the implemented RMS z-score method. This method gives the activity relative to the mean of the signal, for every trial, as a positive number. To illustrate this method, we used an intracranial in vivo recording of an anesthetized rat that is being electrically stimulated every 3 seconds. For each trial, i, which initiates with a stimulus, the root mean square is calculated, then the mean and the standard deviation are obtained in order to determine the z-score value. These steps are repeated for each of the n trials, and then represented in a 3D image. Figure S2. Algorithm flowchart for the implemented PACmethod. The steps for calculating this phase–amplitude plot can be followed for a single channel or for the interaction of two channels, by choosing the inputs as a single or two separate channels. In this case to illustrate the method, we used an artificial modulated signal to show a pure modulation. For every low-frequency band, i, the signal is filtered, and the Hilbert transform is used to determine the phase. For every fast-frequency window, k, the signal is filtered, and the Hilbert transform is used to determine the amplitude. A composite phase–amplitude time seriesis calculated to obtain the mean amplitude distribution over phase bins, and the MI is obtained by normalizing the average for every pair of frequency windows. These steps are repeated for each of the m low-frequency windows and the n high-frequency windows, and are represented in an n by m comodulogram. Figure S3. Algorithm flowchart for the implemented PACmethod. This method can be used to measure the modulation in a single channel or for the interaction of two channels. The signals are obtained in this case from two different channelsof an intracranial in vivo recording of an anesthetized rat. The modulating signal from a chosen channelis filtered in a single modulating band; a peak detection algorithm is used to determine the local minima and maxima in the band range. Local minima and maxima are used to determine the duration of the cycles, thus determining several time windows) for which the wavelet transform of the raw modulated data is calculated for the modulated channel. Using the Hilbert transform, the phase of the modulating cycles is determined to obtain the mean power in the modulated band over phase bins. The power is the average of the entire modulated band, and an average composite phase–amplitude time seriesis calculated. Figure S4. Algorithm flowchart for the implemented PPC method. For each channeland motor cortex), m windows of time are determined, and the Morlet wavelet transform is calculated for each window in both channels [ and ]. For each time window, n frequency windows are determined, and their phasesare calculated. The difference between the two phases [] is measured for every time–frequency epoch and this is used to calculate the phase-coherence value using the formula , which is represented in an n by m matrix.

## Data Availability

The present open-source software can be found in the Github Repository at https://github.com/gucecile/BOARD_FTD_PACC. This also contains the following files derived from in vivo hippocampus recordings for testing: 17,223,000.abf (recording without stimulation), 17,308,005.abf (with a single stimulus 1.5 mA) and 17,308,009.abf (with a double stimulation 1 mA, interval of 0.1 s).

## References

[1] Tudor M, Tudor L, Tudor KI (2005). Hans Berger (1873–1941)–the history of electroencephalography. Acta Med Croatica.

[2] Churchland PS, Sejnowski TJ (2016). Blending computational and experimental neuroscience. Nat Rev Neurosci.

[3] Köster M, Martens U, Gruber T (2019). Memory entrainment by visually evoked theta-gamma coupling. Neuroimage.

[4] Lalo E, Gilbertson T, Doyle L (2007). Phasic increases in cortical beta activity are associated with alterations in sensory processing in the human. Exp Brain Res.

[5] Osipova D, Hermes D, Jensen O (2008). Gamma power is phase-locked to posterior alpha activity. PLoS ONE.

[6] Bruns A, Eckhorn R (2004). Task-related coupling from high- to low-frequency signals among visual cortical areas in human subdural recordings. Int J Psychophysiol.

[7] Vanhatalo S, Palva JM, Holmes MD (2004). Infraslow oscillations modulate excitability and interictal epileptic activity in the human cortex during sleep. Proc Natl Acad Sci USA.

[8] Canolty RT, Edwards E, Dalal SS (2006). High gamma power is phase-locked to theta oscillations in human neocortex. Science.

[9] Penny WD, Duzel E, Miller KJ, Ojemann JG (2008). Testing for nested oscillation. J Neurosci Methods.

[10] Samaha J, Cohen MX (2022). Power spectrum slope confounds estimation of instantaneous oscillatory frequency. Neuroimage.

[11] Tort ABL, Komorowski R, Eichenbaum H, Kopell N (2010). Measuring phase-amplitude coupling between neuronal oscillations of different frequencies. J Neurophysiol.

[12] Voytek B, D’Esposito M, Crone N, Knight RT (2013). A method for event-related phase/amplitude coupling. Neuroimage.

[13] Varela F, Lachaux JP, Rodriguez E, Martinerie J (2001). The brainweb: phase synchronization and large-scale integration. Nat Rev Neurosci.

[14] Palva JM, Palva S, Kaila K (2005). Phase synchrony among neuronal oscillations in the human cortex. J Neurosci.

[15] Fries P (2005). A mechanism for cognitive dynamics: neuronal communication through neuronal coherence. Trends Cogn Sci.

[16] Siegel M, Warden MR, Miller EK (2009). Phase-dependent neuronal coding of objects in short-term memory. Proc Natl Acad Sci USA.

[17] Gregoriou GG, Gotts SJ, Zhou H, Desimone R (2009). High-frequency, long-range coupling between prefrontal and visual cortex during attention. Science.

[18] Delorme A, Makeig S (2004). EEGLAB: an open source toolbox for analysis of single-trial EEG dynamics including independent component analysis. J Neurosci Methods.

[19] Martinez-Cancino R, Delorme A, Kreutz-Delgado K, Makeig S (2020) Computing Phase Amplitude Coupling in EEGLAB: PACTools. In: 2020 IEEE 20th International Conference on Bioinformatics and Bioengineering (BIBE). IEEE, Cincinnati, pp 387–394

[20] Oostenveld R, Fries P, Maris E, Schoffelen J-M (2011). FieldTrip: Open source software for advanced analysis of MEG, EEG, and invasive electrophysiological data. Comput Intell Neurosci.

[21] Tadel F, Baillet S, Mosher JC (2011). Brainstorm: a user-friendly application for MEG/EEG analysis. Comput Intell Neurosci.

[22] Gramfort A, Luessi M, Larson E (2014). MNE software for processing MEG and EEG data. Neuroimage.

[23] Combrisson E, Nest T, Brovelli A (2020). Tensorpac: an open-source Python toolbox for tensor-based phase-amplitude coupling measurement in electrophysiological brain signals. PLoS Comput Biol.

[24] Rothman JS, Silver RA (2018). NeuroMatic: an integrated open-source software toolkit for acquisition, analysis and simulation of electrophysiological data. Front Neuroinform.

[25] Kim YG, Shin JJ, Kim SJ (2021). Minhee analysis package: an integrated software package for detection and management of spontaneous synaptic events. Mol Brain.

[26] Makeig S, Debener S, Onton J, Delorme A (2004). Mining event-related brain dynamics. Trends Cogn Sci.

[27] Väisänen O, Malmivuo J (2009). Improving the SNR of EEG generated by deep sources with weighted multielectrode leads. J Physiol Paris.

[28] Cong F, Ristaniemi T, Lyytinen H (2015). Advanced signal processing on brain event-related potentials: filtering erps in time, frequency and space domains sequentially and simultaneously.

[29] Oppenheim AV, Willsky AS, Nawab SH (1997). Signals and systems.

[30] Cooley JW, Tukey JW (1965). An algorithm for the machine calculation of complex Fourier series. Math Comp.

[31] Sheikh SA, Majoka AZ, Rehman KU (2015). Nonparametric spectral estimation technique to estimate dominant frequency for atrial fibrillation detection. JSIP.

[32] Wang R, Wang J, Yu H (2015). Power spectral density and coherence analysis of Alzheimer’s EEG. Cogn Neurodyn.

[33] Cohen MX (2014). Analyzing neural time series data: theory and practice.

[34] Akin M (2002). Comparison of wavelet transform and FFT methods in the analysis of EEG signals. J Med Syst.

[35] Akujuobi CM (2022). Wavelets and wavelet transform systems and their applications: a digital signal processing approach.

[36] Mallat SG (2009). A wavelet tour of signal processing: the sparse way.

[37] Cohen MX (2019). A better way to define and describe Morlet wavelets for time-frequency analysis. Neuroimage.

[38] Figueiredo A, Nave M, EFDA-JET contributors (2004). Time–frequency analysis of nonstationary fusion plasma signals: a comparison between the Choi–Williams distribution and wavelets. Rev Sci Instrum.

[39] Belluscio MA, Mizuseki K, Schmidt R (2012). Cross-frequency phase-phase coupling between theta and gamma oscillations in the hippocampus. J Neurosci.

[40] Hyafil A, Giraud A-L, Fontolan L, Gutkin B (2015). Neural cross-frequency coupling: connecting architectures, mechanisms, and functions. Trends Neurosci.

[41] Le Van QM, Bragin A (2007). Analysis of dynamic brain oscillations: methodological advances. Trends Neurosci.

[42] Nokia MS, Penttonen M (2022). Rhythmic Memory Consolidation in the Hippocampus. Front Neural Circuits.

[43] Aru J, Aru J, Priesemann V (2015). Untangling cross-frequency coupling in neuroscience. Curr Opin Neurobiol.

[44] Buzsáki G, Wang X-J (2012). Mechanisms of gamma oscillations. Annu Rev Neurosci.

[45] Canolty RT, Knight RT (2010). The functional role of cross-frequency coupling. Trends Cogn Sci.

[46] Colgin LL, Moser EI (2010). Gamma oscillations in the hippocampus. Physiology (Bethesda).

[47] Gauthier-Umaña C, Muñoz-Cabrera J, Valderrama M (2020). Acute effects of two different species of amyloid-β on oscillatory activity and synaptic plasticity in the commissural CA3-CA1 circuit of the hippocampus. Neural Plast.

[48] Boashash B, Azemi G, O’Toole JM (2013). Time-frequency processing of nonstationary signals: advanced TFD design to aid diagnosis with highlights from medical applications. IEEE Signal Process Mag.

[49] Boashash B, Ouelha S (2016). Automatic signal abnormality detection using time-frequency features and machine learning: a newborn EEG seizure case study. Knowl-Based Syst.

[50] Alazrai R, Al-Rawi S, Alwanni H, Daoud MI (2019). Tonic cold pain detection using Choi–Williams time-frequency distribution analysis of EEG signals: a feasibility study. Appl Sci.

[51] Karlsson S, Yu J, Akay M (2000). Time-frequency analysis of myoelectric signals during dynamic contractions: a comparative study. IEEE Trans Biomed Eng.

[52] Jurkiewicz GJ, Hunt MJ, Żygierewicz J (2021). Addressing pitfalls in phase-amplitude coupling analysis with an extended modulation index toolbox. Neuroinformatics.

[53] Dastgheyb RM, Yoo S-W, Haughey NJ (2020). MEAnalyzer—a spike train analysis tool for multi electrode arrays. Neuroinformatics.

[54] Lopez-Calderon J, Luck SJ (2014). ERPLAB: an open-source toolbox for the analysis of event-related potentials. Front Hum Neurosci.

[55] Mørup M, Hansen LK, Arnfred SM (2007). ERPWAVELAB a toolbox for multi-channel analysis of time-frequency transformed event related potentials. J Neurosci Methods.

[56] Peyk P, De Cesarei A, Junghöfer M (2011). ElectroMagnetoEncephalography software: overview and integration with other EEG/MEG toolboxes. Comput Intell Neurosci.

[57] Bao FS, Liu X, Zhang C (2011). PyEEG: an open source Python module for EEG/MEG feature extraction. Comput Intell Neurosci.

[58] Navarrete M, Alvarado-Rojas C, Le Quyen Van M, Valderrama M (2016). RIPPLELAB: a comprehensive application for the detection, analysis and classification of high frequency oscillations in electroencephalographic signals. PLoS ONE.

[59] Lepage KQ, Fleming CN, Witcher M, Vijayan S (2021). Multitaper estimates of phase-amplitude coupling. J Neural Eng.

[60] Hülsemann MJ, Naumann E, Rasch B (2019). Quantification of phase-amplitude coupling in neuronal oscillations: comparison of phase-locking value, mean vector length, modulation index, and generalized-linear-modeling-cross-frequency-coupling. Front Neurosci.

