# Supplementary material for: BOARD-FTD-PACC: a graphical user interface for the synaptic and cross-frequency analysis derived from neural signals
Source: Brain Inform. 2023 May 8;10(1):12. doi: 10.1186/s40708-023-00191-x (PMC10167074; doi:10.1186/s40708-023-00191-x)
Supplement: Supplementary file 1 — Additional file 1: Figure S1. Algorithm flowchart for the implemented RMS z-score method. This method gives the activity relative to the mean of the signal, for every trial, as a positive number. To illustrate this method, we used an intracranial in vivo recording of an anesthetized rat that is being electrically stimulated every 3 seconds. For each trial, i, which initiates with a stimulus, the root mean square is calculated, then the mean and the standard deviation are obtained in order to determine the z-score value. These steps are repeated for each of the n trials, and then represented in a 3D image. Figure S2. Algorithm flowchart for the implemented PACmethod. The steps for calculating this phase–amplitude plot can be followed for a single channel or for the interaction of two channels, by choosing the inputs as a single or two separate channels. In this case to illustrate the method, we used an artificial modulated signal to show a pure modulation. For every low-frequency band, i, the signal is filtered, and the Hilbert transform is used to determine the phase. For every fast-frequency window, k, the signal is filtered, and the Hilbert transform is used to determine the amplitude. A composite phase–amplitude time seriesis calculated to obtain the mean amplitude distribution over phase bins, and the MI is obtained by normalizing the average for every pair of frequency windows. These steps are repeated for each of the m low-frequency windows and the n high-frequency windows, and are represented in an n by m comodulogram. Figure S3. Algorithm flowchart for the implemented PACmethod. This method can be used to measure the modulation in a single channel or for the interaction of two channels. The signals are obtained in this case from two different channelsof an intracranial in vivo recording of an anesthetized rat. The modulating signal from a chosen channelis filtered in a single modulating band; a peak detection algorithm is used to determine the local minima a [file 40708_2023_191_MOESM1_ESM.docx]

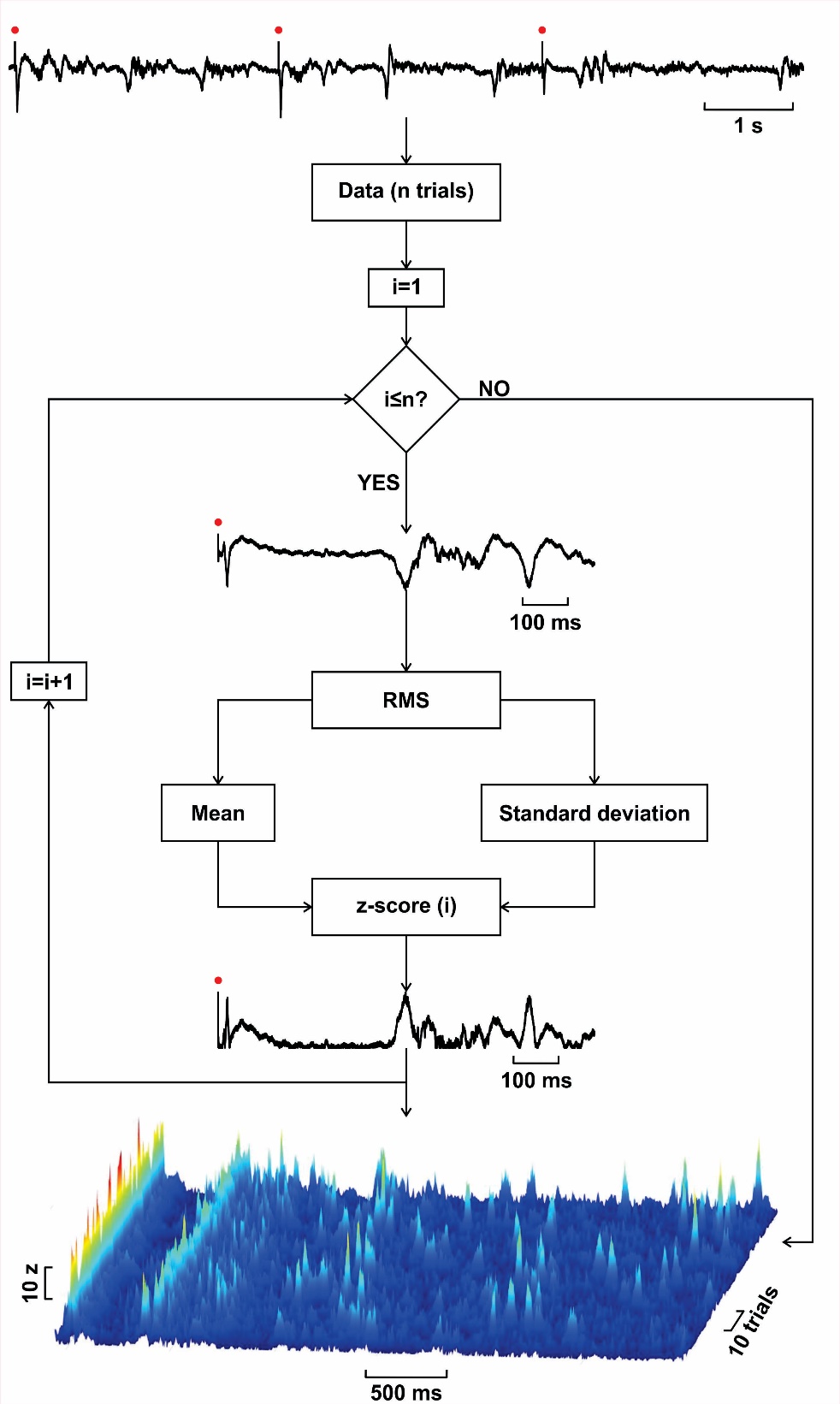


**Figure S1. Algorithm flowchart for the implemented RMS z-score method.** This method gives the activity relative to the mean of the signal, for every trial, as a positive number. To illustrate this method, we used an intracranial *in vivo* recording of an anesthetized rat that is being electrically stimulated every 3 seconds. For each trial, *i*, which initiates with a stimulus (red dot), the root mean square is calculated, then the mean and the standard deviation are obtained in order to determine the z-score value. These steps are repeated for each of the *n* trials, and then represented in a 3D image.


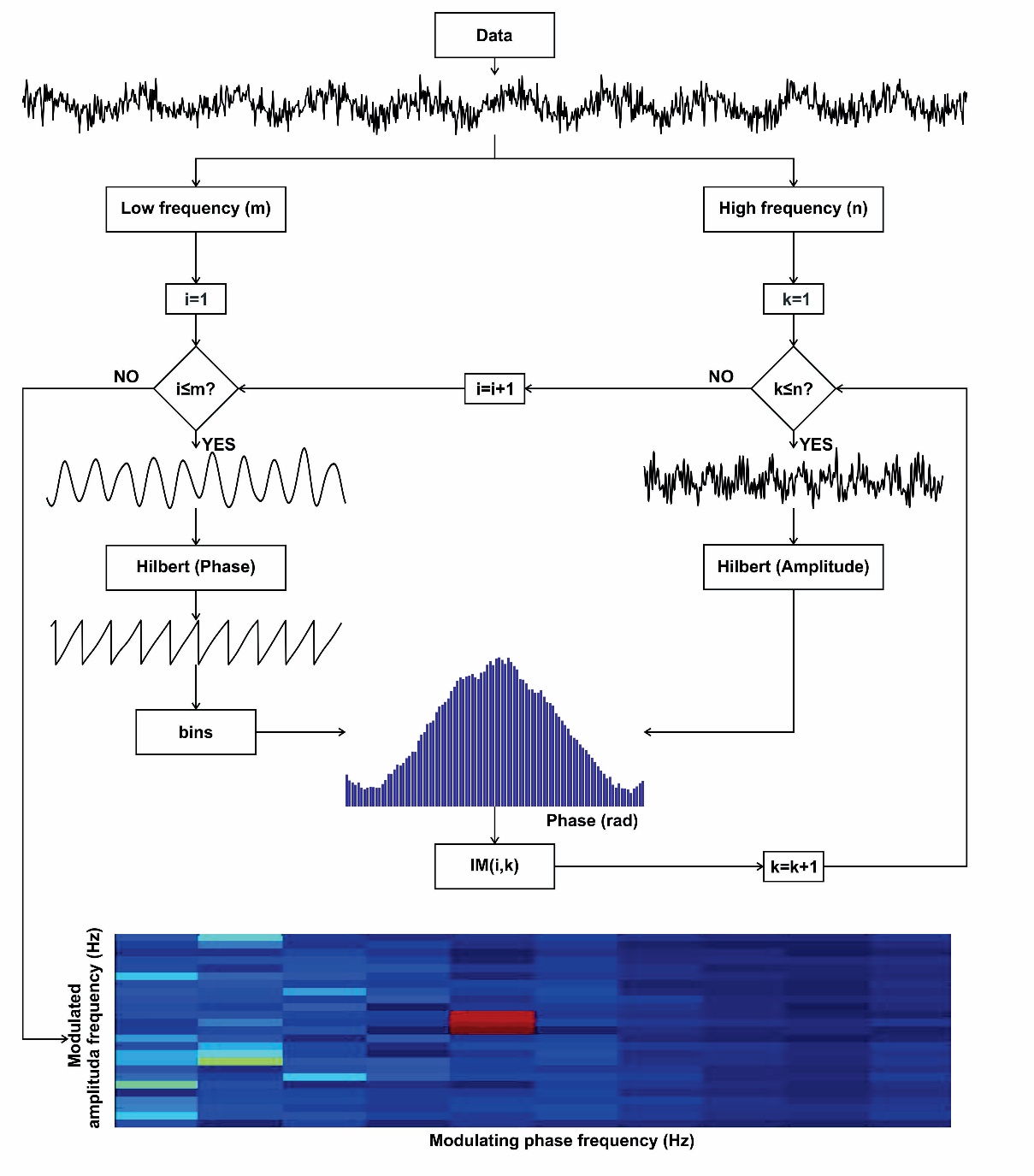


**Figure S2. Algorithm flowchart for the implemented PAC (comodulogram) method.** The steps for calculating this phase-amplitude plot can be followed for a single channel or for the interaction of two channels, by choosing the inputs as a single or two separate channels. In this case to illustrate the method, we used an artificial modulated signal to show a pure modulation. For every low frequency band, *i*, the signal is filtered, and the Hilbert transform is used to determine the phase. For every fast frequency window, *k,* the signal is filtered, and the Hilbert transform is used to determine the amplitude. A composite phase-amplitude time series (fp, AfA) is calculated to obtain the mean amplitude distribution over phase bins, and the MI is obtained by normalizing the average for every pair of frequency windows. These steps are repeated for each of the *m* low frequency windows and the *n* high frequency windows, and are represented in an *n* by *m* comodulogram.


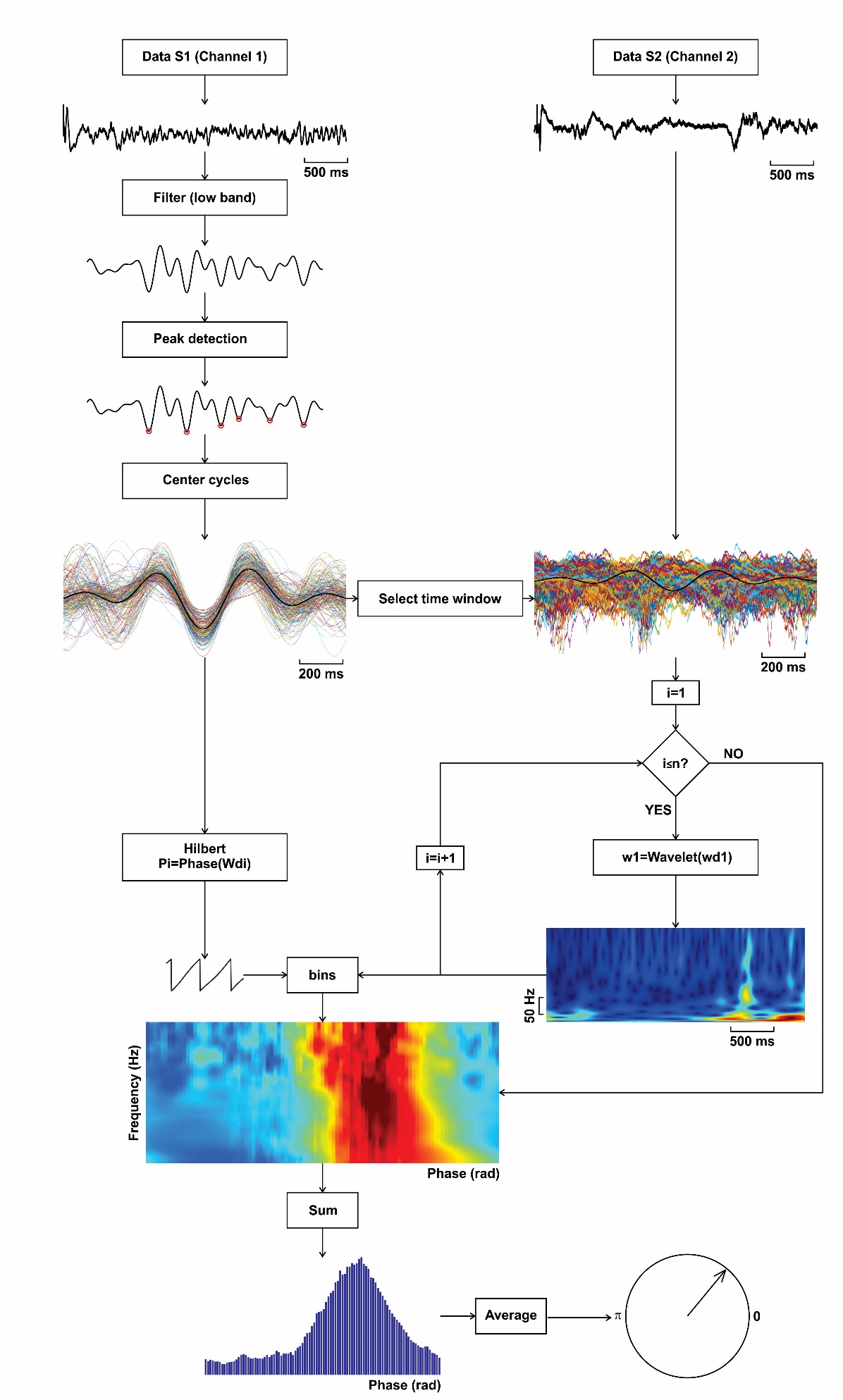


**Figure S3. Algorithm flowchart for the implemented PAC (cycle detection) method.** This method can be used to measure the modulation in a single channel or for the interaction of two channels. The signals are obtained in this case from two different channels (thalamus S1 and motor cortex S2) of an intracranial *in vivo* recording of an anesthetized rat. The modulating signal from a chosen channel (in this case thalamus S1) is filtered in a single modulating band; a peak detection algorithm is used to determine the local minima and maxima in the band range. Local minima and maxima are used to determine the duration of the cycles, thus determining several time windows (wd(i)) for which the wavelet transform of the raw modulated data is calculated for the modulated channel (in this case motor cortex S2). Using the Hilbert transform, the phase of the modulating cycles is determined to obtain the mean power in the modulated band over phase bins. The power is the average of the entire modulated band, and an average composite phase-amplitude time series (fp, AfA) is calculated.


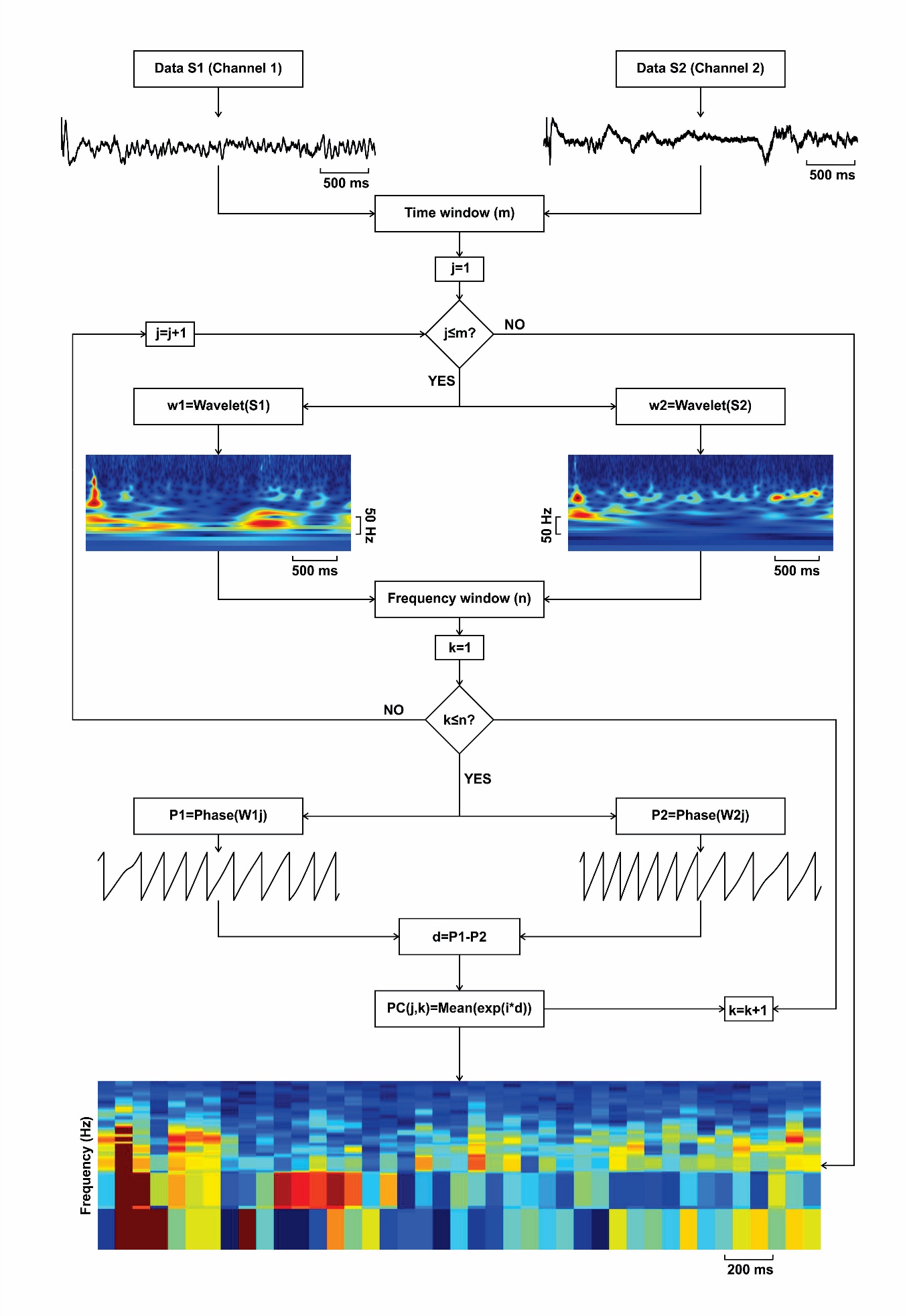
**Figure S4. Algorithm flowchart for the implemented PPC method.** For each channel (thalamus ($S1$) and motor cortex ($S2$)), *m* windows of time are determined, and the morlet wavelet transform is calculated for each window in both channels [$W_{1} (j)$ and $W_{2} (j)$]. For each time window, *n* frequency windows are determined, and their phases ($P_{1} \left( j \right)$ and $P_{2} (j)$) are calculated. The difference between the two phases [${d= P}_{1} \left( j \right)-P_{2} (j)$] is measured for every time-frequency epoch and this is used to calculate the phase-coherence value using the formula $PC\left( j,k \right)=mean(e^{id})$, which is represented in an *n* by *m* matrix.
